# Primary implant stability of two implant macro-designs in different alveolar ridge morphologies: an in vitro study

**DOI:** 10.1186/s40729-025-00605-x

**Published:** 2025-03-06

**Authors:** Anna Jenner, Gabriela P. Sabatini, Samir Abou-Ayash, Emilio Couso-Queiruga, Vivianne Chappuis, Clemens Raabe

**Affiliations:** 1 Department of Oral Surgery and Stomatology, School of Dental Medicine, University of Bern, Bern, Switzerland; 2 Department of Prosthodontics, University of São Paulo, São Paulo, Brazil; 3 Department of Reconstructive Dentistry and Gerodontology, School of Dental Medicine, University of Bern, Bern, Switzerland; 4 Department of Prosthetic Dentistry and Material Science, University Medical Center of the Johannes Gutenberg University Mainz, Mainz, Germany; 5 Department of Oral Surgery and Implantology, Goethe University, Theodor-Stern-Kai 7, 60596 Carolinum, Frankfurt am Main, Germany

**Keywords:** Dental implants; single tooth, Clinical decision-making, Image-guided surgery, Tooth extraction, Alveolar ridge, Resonance frequency analysis, Torque

## Abstract

**Purpose:**

The primary aim of this in vitro study was to investigate the primary implant stability obtained in immediate and late implant placement scenarios. Secondary aims evaluated the effect of two distinct implant macro-designs and examined the correlation between resonance frequency analysis (RFA) and final insertion torque.

**Methods:**

Partially edentulous maxillary models including six single sites simulating extraction sockets and healed alveolar ridges were used. Virtual implant planning facilitated static computer-assisted implant placement of bone level implants with either a shallow-threaded and cylindrical (BL), or deep-threaded and tapered implant macro-design (BLX). The insertion torque was continuously measured during implant placement, and RFA was performed after final implant positioning.

**Results:**

One-hundred and forty-four implants were equally distributed to two alveolar ridge morphologies and implant designs. Higher final insertion torque and RFA values were observed for implants placed in healed ridges compared to extraction sockets (40.8 ± 13.5 vs. 20.6 ± 8.4 Ncm, and RFA 70.7 ± 2.8 vs. 59.6 ± 6.5, both *p* < 0.001), and for BL implants compared to BLX implants (35.7 ± 13.0 vs. 25.7 ± 8.9 Ncm, and RFA 66.7 ± 4.4 vs. 63.6 ± 4.9, both *p* < 0.001). Insertion torque and mean RFA values positively correlated (*r* = 0.742; *p* < 0.001).

**Conclusion:**

Primary implant stability is significantly affected by the alveolar ridge morphology and the implant macro-design, demonstrating higher values in healed sites and shallow-threaded, cylindrical implants. Therefore, a tailored selection of the implant design depending on the implant placement and loading protocol is recommended.

## Background

Dental implants are a well-established and reliable option for replacing missing teeth in both partially and fully edentulous patients. The stability of dental implants and their long-term success are ensured through the process of osseointegration [1]. Osseointegration refers to the direct structural and functional connection between living bone and the surface of a load-bearing implant [2]. This process requires achieving primary stability at the time of implant placement, followed by undisturbed wound healing, facilitating a series of critical biological events that culminate in osseointegration and peri-implant tissue stability [3].

Primary implant stability during placement is attained through the direct mechanical engagement with the surrounding alveolar bone [4]. Over the course of 4 to 8 weeks, primary stability is gradually superseded by secondary stability, which is driven by a biological bone remodeling around the implant [5–7]. Insufficient primary stability may jeopardize the process of osseointegration, as micromovements between implant and surrounding bone exceeding 100 μm potentially disrupt bone healing and lead to fibrous encapsulation rather than osseointegration [4, 8–11].

Comprehensive treatment planning for failing teeth and dental implant therapy is complex, encompassing numerous factors such as the choice of the ideal implant design characteristics, the appropriate timing of implant placement following tooth extraction and the subsequent loading protocols. The selection of these treatment options should aim to predictably achieve long-term treatment success, including optimal esthetic outcomes and a low risk of complications, while also striving to reduce the number of surgical and clinical procedures, whenever feasible [12, 13]. As patient interest in shorter treatment times continues to grow, immediate implant placement has gained popularity, particularly when paired with immediate restoration, with or without immediate loading [14–17]. However, the success of immediate protocols depends significantly on achieving high primary stability at the time of implant placement, which is often challenged by local morphological factors when comparing implant engagement in fresh extraction sockets versus late implant placement in healed alveolar ridges [17]. Several additional factors also influence primary implant stability, including alveolar bone density and dimensions, implant design characteristics, and surgical technique [18–22]. Although the precise threshold of adequate primary stability for immediate restoration or loading remains unclear, a minimum insertion torque of 35 Ncm during implant placement is frequently recommended [7, 17, 23].

To address this challenge of adequate primary stability, particularly in immediate implant placement scenarios, implants with modified macro-designs have been developed in recent years. These modifications, which include changes to implant shape, surface topography, and thread design (depth, pitch, and shape), are intended to enhance primary stability [4, 8, 10]. While a recent review suggested only minimal differences in primary stability between tapered and non-tapered implants [24], multiple in vitro and in vivo studies indicate that tapered designs generally provide higher primary stability compared to cylindrical implants [25–27]. Despite these findings, there is only limited information on the effect of alveolar ridge morphology on primary implant stability and the influence of various implant macro-designs. Consequently, there is a need for recommendations on selecting specific implant specifications tailored to different clinical scenarios involving immediate placement and loading protocols.

Therefore, the primary aim of this in vitro investigation was to assess the influence of two different alveolar ridge morphologies on the primary implant stability. The secondary aim was to assess the impact of two implant macro-designs on primary stability and to examine the reliability of resonance frequency analysis (RFA) in comparison to final insertion torque as a measure for primary implant stability. The null hypotheses were as follows: alveolar ridge morphology (H01), implant macro-design (H02), and their interactions (H03) do not influence primary implant stability during implant placement.

## Methods

### Models and virtual implant planning

**Fig. 1:**
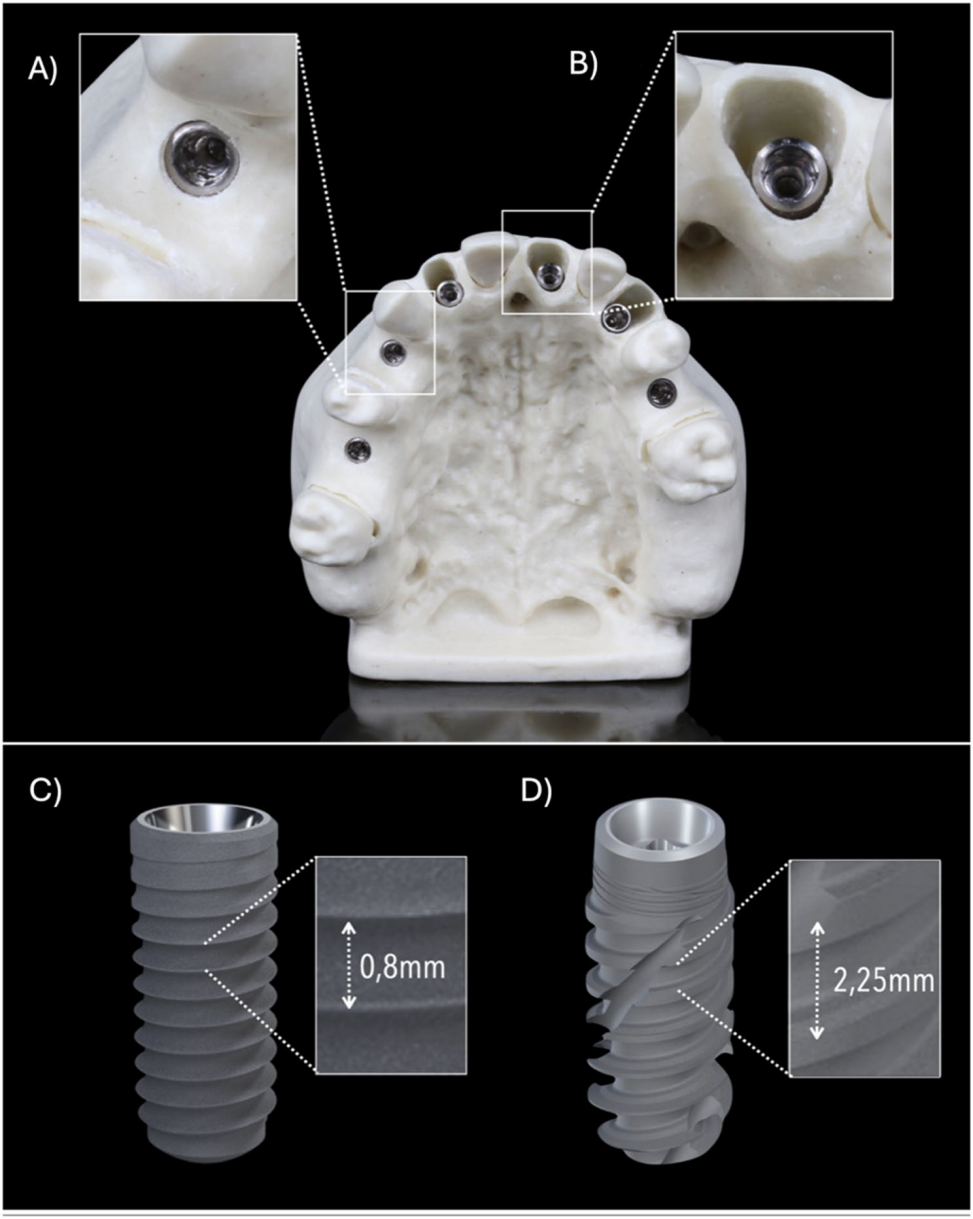
Representative images of the study variables. Representation of different alveolar ridge morphologies: Healed ridge morphology (**A**) and fresh extraction socket morphology (**B**). Representation of different implants used in the study, with different thread pitch distances: BL 4.1 × 12 mm RC (**C**) and BLX 4 × 12 mm RB (**D**)

The present in vitro study was designed and conducted in the Department of Oral Surgery and Stomatology at the University of Bern, Switzerland from November 2021 to February 2022. Standardized partially edentulous models mimicking a cortico-spongious alveolar bone density D2 were used (BoneModels, Castellón de la Plana, Spain) [28]. Each model presented six single-tooth edentulous sites corresponding to the FDI teeth positions 16, 14 and 25 simulating healed alveolar ridge morphologies, and to the FDI teeth positions 12, 21 and 23 simulating fresh extraction sockets (Fig. 1). For each model, a virtual implant planning was performed in a dedicated software package (coDiagnostiX 10.5, Dental Wings Inc, Montreal, Canada) based on a Cone Beam Computed Tomography (CBCT) scan (8 × 5 cm, 80 μm voxel size, 90kVp, 1mAs; 3D Accuitomo 170, J. Morita Corp, Osaka, Japan) and a surface scan using a laboratory scanner (3Shape 4, 3Shape Inc, Copenhagen, Denmark). After superimposing the files, the ideal 3D implant position for each site was planned based on a digital wax-up (Zirkonzahn. Modellier, Zirkonzahn GmbH, Gaus, Italy) for screw-retained single implant crowns by an experienced clinician (C.R). In extraction socket sites, an apical implant engagement of at least 4 mm was respected. Subsequently, the surgical guide was designed with a material thickness of 3.5 mm and a guide-to-tooth offset of 0.15 mm. Multiple fenestrations were included to allow for a visual verification of the guides fit on the model. The guides were manufactured for each model using a transparent, light-cured resin for stereolithography (ProArt Print Splint, Ivoclar Vivadent AG, Schaan, Lichtenstein) in a 3D printer (PrograPrint PR5, Ivoclar Vivadent AG, Schaan, Lichtenstein).

### Guided implant placement and study groups

To recreate the clinical scenario as closely as possible, the models were mounted in phantom-heads. Afterwards, fully guided sCAIS procedures according to the manufacturer’s protocols were carried out using a surgical motor (iChiropro, Bien-Air, Bienne, Switzerland).

The study involved two bone-level type implants, each with distinct macro-design features (Fig. 1):

- Shallow-threaded parallel-walled implant body with a thread pitch of 0.8 mm (BL 4.1 × 12 mm RC, Straumann AG. Basel, Switzerland), representing a conventional design available for decades to address a broad range of clinical indications, and
- Deep-threaded tapered implant body with a thread pitch of 2.25 mm (BLX 4.0 × 12 mm RB, Straumann AG. Basel, Switzerland), a recently introduced design intended to achieve high primary stability, particularly in immediate implant placement protocols.

These implants were randomly assigned to the edentulous sites, ensuring equal sample sizes for each group.

### Measurement of primary implant stability

**Fig. 2:**
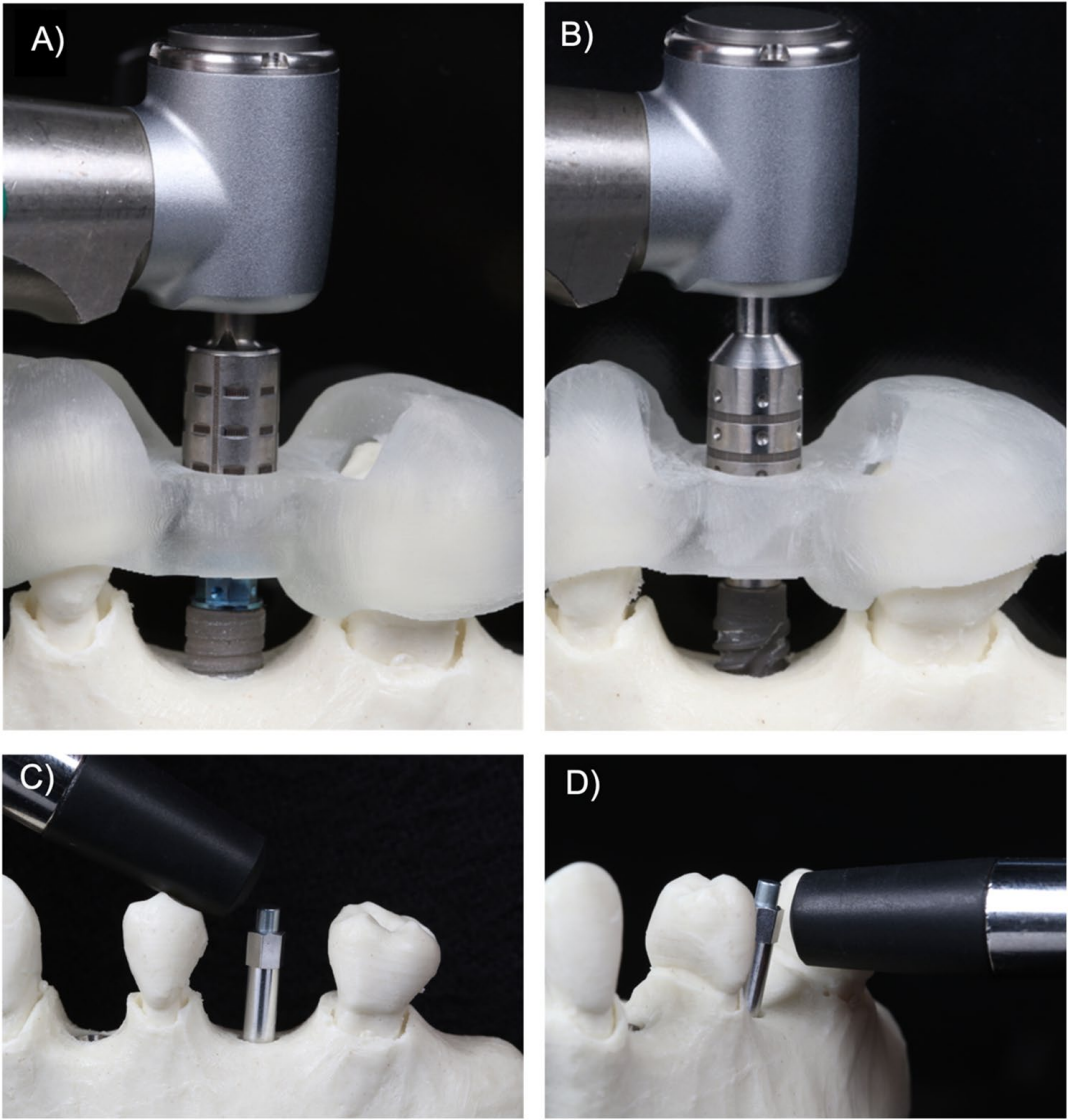
Implant placement procedure of the BL (**A**) and BLX implant (**B**). Resonance frequency analysis using the Osstell device in mesio-distal direction (**C**) and bucco-lingual direction (**D**)

The primary stability of all the implants was assessed using the following two methods:

- Continuous measurement of the insertion torque (Ncm) over time during implant placement using the surgical motor (iChiropro, Bien-Air, Bienne, Switzerland); and
- Resonance Frequency Analysis (RFA) after final implant placement using hand-tightened implant-specific transducers and a RFA device (Osstell ISQ, Integration Diagnostics Ltd., Goteborgsvagen, Sweden). The RFA assessment was conducted three times in both the mesio-distal and bucco-lingual orientations, recording the lowest value from each orientation. The mean of these two lowest values was then calculated (Fig. 2).

### Statistical analysis

The primary outcome of the present study was the comparison of final torque values between the different alveolar ridge morphologies, followed by the secondary outcome of the same variables for the different implant macro-designs. Finally, a correlation between RFA and final torque values was investigated. All collected data was presented as mean and standard deviation (SD). Two-way analysis of variance (ANOVA) was used for the primary and secondary outcomes to verify the effects of the independent variables (alveolar ridge morphology and implant macro-design) on the dependent variables (torque and mean RFA). Main and interaction effects were tested, and multiple comparisons used Sidak’s post hoc. Effect sizes and observed power were calculated, and interaction plots were designed. The correlation between torque and mean RFA was performed using Pearson’s bivariate correlation coefficient. All the analyses were carried out using IBM SPSS v.26 software, adopting a significance level of 5%.

## Results

### Study sample

A total of 144 implants (BL *n* = 72, BLX *n* = 72) were equally distributed to static computer-assisted implant placement in single tooth sites with healed alveolar ridge (*n* = 72) or extraction socket morphology (*n* = 72) in 36 models.

### Alveolar ridge morphology

**Fig. 3:**
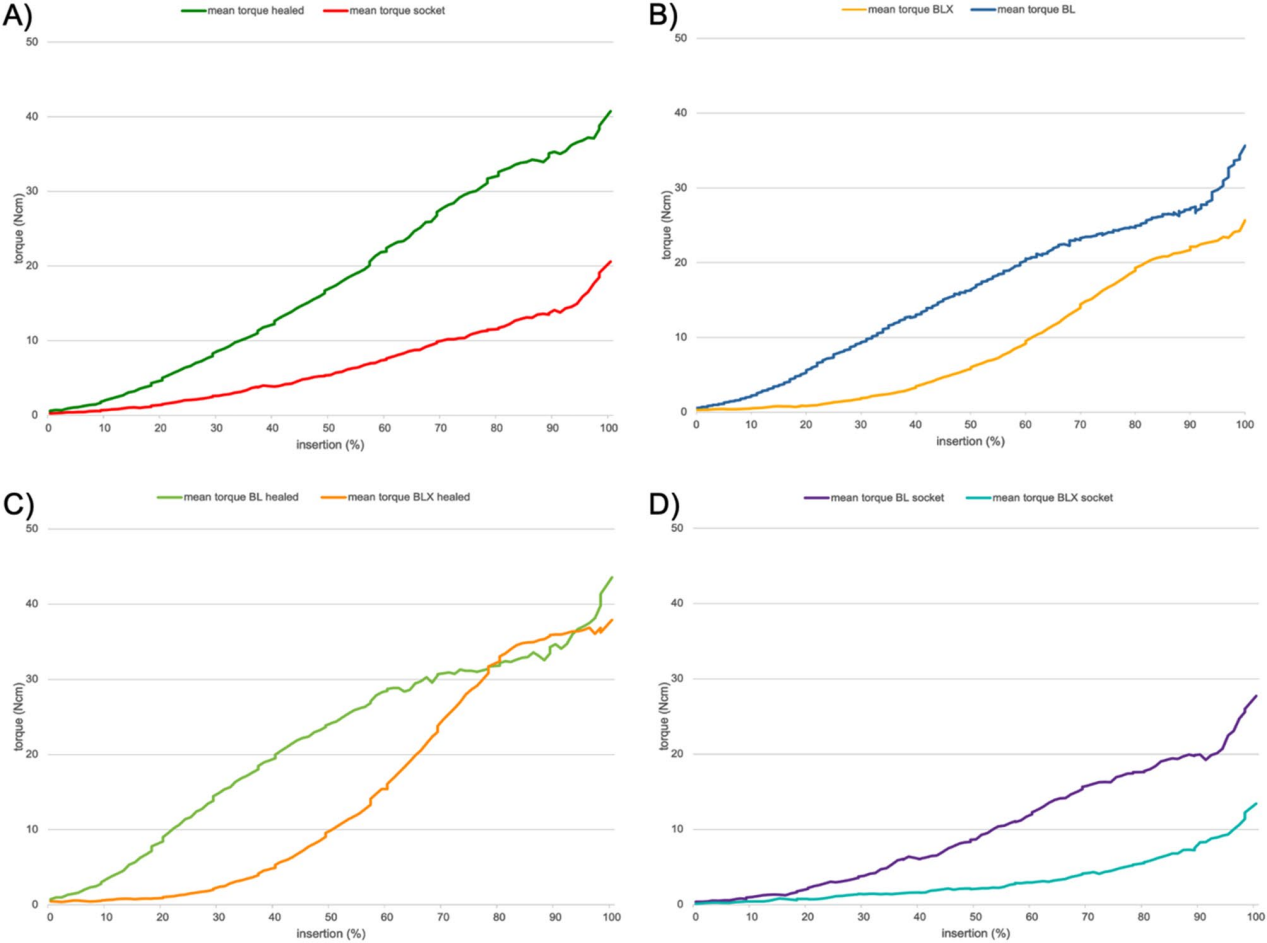
Mean torque buildup during implant insertion for extraction sockets vs. fully healed ridges overall implant macro-designs (**A**), BL vs. BLX implants overall alveolar ridge morphologies (**B**), BL vs. BLX implants in fully healed ridges (**C**), and BL vs. BLX implants in extraction sockets (**D**)

**Fig. 4:**
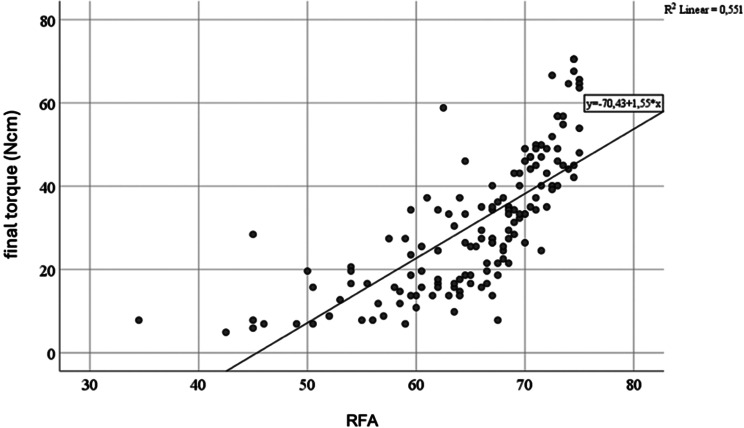
Bivariate Pearson correlation between final torque and RFA

Higher final torque values were observed when implants were placed in healed ridge sites compared to extraction sockets (*p* < 0.001). Notably, the insertion torque increased linearly, with a steeper incline in healed ridge sites compared to extraction sockets (Fig. 3). Similarly, higher mean RFA values were observed for implants in healed ridges compared to extraction sockets (*p* < 0.001). A positive and statistically significant correlation was found between final insertion torque and mean RFA values (*r* = 0.742; *p* < 0.001) as illustrated in Fig. 4.

**Fig. 5:**
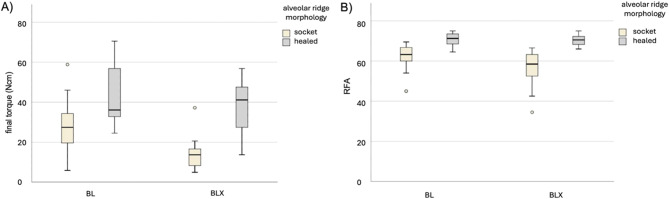
Effects of alveolar ridge morphology and implant design on final torque (**A**) and RFA (**B**)

Descriptive statistics and corresponding box plots are displayed in Table 1; Fig. 5. The main effects and multiple comparisons between implant type and alveolar ridge morphology for mean RFA and final torque are presented in Tables 2 and 3.

**Table 1:** Multiple comparison between implant type and alveolar ridge morphology for mean final torque and RFA

	BL + BLX		BL		BLX	
	*n*	mean ± SD	*n*	mean ± SD	*n*	mean ± SD
**Final torque (Ncm)**						
socket + healed	144	30.7 ± 11.0	72	35.7 ± 13.0	72	25.7 ± 8.9
socket	72	20.6 ± 8.4	36	27.7 ± 10.9	36	13.4 ± 5.9
healed	72	40.8 ± 13.5	36	43.6 ± 15.1	36	37.9 ± 11.9
**Mean RFA**						
socket + healed	144	13.4 ± 4.7	72	66.7 ± 4.4	72	63.6 ± 4.9
socket	72	59.6 ± 6.5	36	62.4 ± 5.7	36	56.8 ± 7.3
healed	72	70.7 ± 2.8	36	70.9 ± 3.0	36	70.4 ± 2.5

**Table 2:** Main effects and interaction for mean final torque and RFA

Effect	*p*-value	effect size	observed power
**Final torque**			
implant type	< 0.001	0.16	1.00
alveolar ridge morphology	< 0.001	0.44	1.00
interaction	0.025	0.04	0.61
**Mean RFA**			
implant type	< 0.001	0.09	0.96
alveolar ridge morphology	< 0.001	0.55	1.00
interaction	0.003	0.06	0.86

**Table 3:** Multiple comparisons between implant type and alveolar ridge morphology for mean RFA and final torque

	Implant type	Alveolar ridge morphology	*p*-value
**Final torque**			
	BLX	socket vs. healed	< 0.001
	BL	socket vs. healed	< 0.001
	BL vs. BLX	socket	< 0.001
	BL vs. BLX	healed	0.037
**Mean RFA**			
	BLX	socket vs. healed	< 0.001
	BL	socket vs. healed	< 0.001
	BL vs. BLX	socket	< 0.001
	BL vs. BLX	healed	0.640

### Implant macro-design

Higher final torque values were observed in BL implants compared to BLX implants (*p* < 0.001). BL implants exhibited a more linear torque increase in healed sites, whereas BLX implants showed a more progressive torque formation curve (Fig. 3). Similarly, higher mean RFA values were recorded for BL implants compared to BLX implants (*p* < 0.001).

Descriptive statistics and corresponding box plots are displayed in Table 1 and Fig. 5. The main effects and multiple comparisons between implant type and alveolar ridge morphology for mean RFA and final insertion torque are illustrated in Tables 2 and 3.

### Interactions

The alveolar ridge morphologies were compared among each implant macro-design group. The BL implants presented statistically significant higher final torque and mean RFA values in healed sites compared to extraction socket sites (*p* < 0.001). Also, in the BLX implant group the results were statistically significant and higher final torque and mean RFA values were observed in healed sites compared to extraction socket sites (*p* < 0.001).

Conversely, the implant macro-design was analyzed according to the alveolar ridge morphologies. When placed in extraction socket sites, the BL implants presented statistically significant higher final torque and mean RFA compared to BLX implants (*p* < 0.001). Single outliers in torque and RFA values were observed in both the BL and BLX groups at socket sites, reflecting the challenging anatomical features that potentially compromise the predictability of primary stability in immediate implant placement (Fig. 5). When placed in healed sites, statistically significant higher final torque values for BL implants compared to BLX implants could be achieved (*p* = 0.037). However, no statistically significant difference was observed between the mean RFA values of BL and BLX implants in fully healed sites (Tables 1 and 2, and Table 3). The interactions of implant type and alveolar ridge morphology had a statistically significant effect on the final torque (*p* = 0.025) and on the mean RFA (*p* = 0.003).

## Discussion

The present in vitro study examined the primary stability of implants with two different macro-designs placed into simulated fresh extraction sockets compared to healed alveolar ridges. The results of this investigation demonstrate higher final torque and RFA values in fully healed compared to extraction socket sites and for BL compared to BLX implants. The final insertion torque and RFA values were positively correlated, demonstrating the reliability of RFA values in implant stability assessment. Therefore, H01, H02, and H03 were rejected.

The present study demonstrates that the morphology of the alveolar ridge significantly impacts primary implant stability, with extraction sockets demonstrating lower final implant insertion torque and RFA values compared to healed alveolar ridges. This is in line with the results from a clinical trial, reporting insertion torques of 65.5 Ncm versus 53.7 Ncm and RFA values of 72.8 versus 63.9 for healed sites as compared to extraction sockets [29]. Similarly, another in vitro study reported insertion torques of 49 Ncm versus 28 Ncm and RFA values of 62 versus 53 for full embedment in bone compared to circular defects [30]. The significantly lower primary implant stability in extraction socket sites might be attributed to the incomplete embedding in bone [15, 31]. To achieve sufficient primary stability in these cases, it is recommended that the implant osteotomy extend 3–4 mm apically beyond the socket, or modify the drilling protocol, underpreparing the osteotomy [29, 31, 32]. Contrarily, implants in healed alveolar ridges are fully embedded in bone, a factor also contributing to implant positioning accuracy [33]. Significantly higher positional deviations between planned and final implant positions, pointing to the zone of less resistance, were found for extraction socket sites [33, 34]. These deviations may affect apical implant engagement and, consequently, primary implant stability [33–35]. While higher primary implant stability values are a prerequisite for immediate loading protocols, excessively high insertion torque does not necessarily enhance the process of osseointegration [36–38]. In fact, high insertion torques could induce pronounced local bone necrosis, potentially compromising osseointegration [25, 39]. Conversely, and in conjunction with conventional implant loading, low insertion torque values do not negatively affect osseointegration as long as implant stability remains above 10 Ncm [7, 37].

In addition to local anatomical characteristics, the macro-design of the implant plays a significant role in achieving primary stability during implant placement [4, 8, 10]. Interestingly, the present study demonstrated lower primary implant stability for BLX implants compared to BL implants across both simulated clinical scenarios. These results are supported by an in vitro study that reported higher RFA and final torque values for BL implants across various bone densities compared to BLX implants [30]. Contrarily, an ex vivo study reported higher RFA and final torque values for BLX implants compared to BL implants in low-density scenarios using cancellous iliac porcine crest blocks [40]. Despite the differences observed in the present study, both implant designs provided sufficient primary stability for conventional loading protocols in extraction sockets, as final torque values exceeded the 10 Ncm threshold [37]. However, neither the BL nor BLX design met the recommended 35 Ncm threshold for immediate loading in this study [17]. Interestingly, in healed sites, the influence of implant design on primary stability was less significant, with both designs potentially qualifying for immediate implant loading protocols. The higher primary implant stability of BL implants may be attributed to their smaller thread pitch compared to BLX implants. A smaller thread pitch increases the implant surface area, leading to greater bone-to-implant contact and enhanced mechanical anchorage [40–43]. Additionally, the core diameter of the BLX implant (3.5 mm) is significantly smaller than that of the BL implant (4.1 mm). Increased implant diameters and non-self-cutting threads are also associated with higher primary implant stability [41, 44, 45]. Conversely, tapered implant body designs have been suggested to achieve higher primary implant stability than cylindrical-shaped implants [25–27, 39, 46, 47]. This is likely due to greater compression of the surrounding bone, which may provide favorable stress on the tissue and reduces the risk of micromovement [25, 41]. Therefore, an under-preparation of the implant bed for tapered BLX implants could potentially result in higher primary stability and might reach the threshold for immediate implant loading. This is supported by a recent randomized controlled study, demonstrating significantly higher primary stability for implants placed in sites with under-preparation compared to those inserted following a conventional drilling sequence [48]. Consequently, the implant specifications, macro-design, and osteotomy protocols should be tailored to the individual site-specific tissue characteristics [7]. The implant designs investigated in this project were suitable for conventional loading protocols in both clinical scenarios, with BL implants consistently demonstrating higher primary stability. However, selecting BLX implants may be advantageous in cases where anatomical restrictions in the apical region of the osteotomy favor the use of a tapered implant design. The findings of this study indicate that achieving primary stability compatible with immediate loading protocols during immediate implant placement was not predictable for either implant design. Therefore, this treatment protocol should be limited to carefully selected cases, with conventional loading recommended in situations where primary stability is uncertain.

Primary implant stability is commonly assessed at the time of placement using insertion torque. However, this method is limited to a single-point measurement, as repeated assessments would disrupt the osseointegration process. RFA offers an alternative, allowing for non-invasive monitoring of implant stability post-placement by providing an Implant Stability Quotient score, ranging from 1 to 100 [49]. In this study, both final insertion torque and RFA values were recorded, and a positive, statistically significant correlation between the two was observed. This finding aligns with prior research from both in vitro and clinical studies, which also report a positive correlation between final insertion torque and RFA values [38, 50–53]. These results support RFA as a reliable tool for evaluating primary implant stability, particularly when compared to insertion torque. However, caution is warranted in long-term monitoring, as conflicting evidence exists regarding the relationship between RFA measurements, marginal bone loss, and other clinical parameters [54].

Several limitations of this study should be acknowledged. First, as an in vitro study, the generalizability of the results is limited, and caution is needed when extrapolating these results to clinical scenarios. The acrylic models used mimic the D2 density of human cortico-spongious bone but do not fully replicate the clinical environment with its complexities at a specific location in the alveolar ridge and in the variety of the different sites throughout the maxilla and mandible. Further, anatomical limitations, such as limited vertical and horizontal bone, can occur in clinical situations and are not considered in this study. This could require bone augmentation procedures or selection of narrower implant diameters and shorter implants, potentially leading to reduced primary implant stability. Second, this study compared two implants with multiple differing macro-design features, potentially obscuring the individual effects of each feature, making it difficult to attribute the outcomes to a specific design characteristic. Additionally, adjustments to implant specifications, such as using longer implants for enhanced apical engagement or wider implants for increased lateral bone engagement would influence the primary stability. Third, only one bone density and drilling protocol were examined, leaving the influence of other factors unclear.

Future studies should explore a broader range of bone densities and include different alveolar ridge morphologies with horizontal and vertical bone defects and their impact on primary implant stability. Further, different surgical techniques should be considered, regarding the potential of reaching the threshold for immediate implant loading in immediate placement procedures. Additionally, investigating implants with singularly distinct macro-design features would provide more clarity. Clinical validation is needed to eradicate the limitations of generalizability and recommended to assess osseointegration and secondary implant stability over time during follow-up periods.

## Conclusion

Within the limitations of this in vitro study, it can be concluded that:

- Implants inserted in healed alveolar ridges show higher final insertion torques and RFA values as compared to fresh extraction sockets.
- BL implants were found to have higher final insertion torques and RFA values compared to BLX implants in both simulated clinical scenarios.
- RFA was shown to be a reliable and repeatable method to assess primary implant stability as compared to the insertion torque values.

## Data Availability

The datasets generated during the current study are available from the corresponding author on reasonable request.

## Abbreviations

3D: Three-dimensional
ANOVA: analysis of variance
BL: Straumann Bone Level implant
BLX: Straumann Bone Level X implant
CBCT: Cone-beam computed tomography
FDI: Fédération dentaire internationale
RFA: Resonance frequency analysis
sCAIS: static computer-assisted implant surgery
SD: Standard deviation
